# Protective effects of Xinji′erkang on myocardial infarction induced cardiac injury in mice

**DOI:** 10.1186/s12906-017-1846-5

**Published:** 2017-06-26

**Authors:** Juan Hu, Yong-xue Zhang, Li Wang, Ling Ding, Guang-yao Huang, Guo-wei Cai, Shan Gao

**Affiliations:** Department of Pharmacology, Basic Medical College, Anhui Medical University, Hefei, 230032 China

**Keywords:** Xinji′erkang, Myocardial infarction, Cardiovascular remodeling, Electrocardiography, Inflammatory cytokines

## Abstract

**Background:**

Myocardial infarction (MI) is a major risk factor responsible for morbidity and mortality. Xinji′erkang (XJEK) has been clinically used as an effective medication in the treatment of coronary heart disease and myocarditis. The purpose of this study was to investigate the cardioprotective effect of Xinji′erkang on MI mice.

**Methods:**

Forty male mice were randomly assigned into four groups as follows (*n* = 10): sham, model, MI with administration of XJEK and fosinopril for four weeks. At the end of studies, hemodynamic parameters and electrocardiography (ECG) were recorded. Heart and body mass were measured and heart weight/body weight (HW/BW) ratio was calculated as index of hypertrophy. The hypertrophy of heart and aorta was examined using the hematoxylin and eosin (HE) staining, and the collagen deposition was evaluated using Van Gieson (VG) staining. Serum nitric oxide level (NO), superoxide dismutase (SOD) activity and malondialdehyde (MDA) concentration were assayed by colorimetric analysis. The expressions of endothelial NO synthetase (eNOS) expression in serum and cardiac tissues were determined using ELISA assay and immunohistochemistry. Angiotensin II (Ang II) in serum and cardiac tissues was measured using ELISA assay. Besides, tumor necrosis factor-α (TNF-α), interleukin1β (IL-1β) and interleukin10 (IL-10) were observed in cardiac tissues with ELISA assay as well.

**Results:**

The administration of XJEK significantly improved cardiac dysfunction and abnormal ECG with reduced HW/BW ratio and ameliorated cardiomyocyte hypertrophy and collagen deposition compared to MI, which was partly due to the decreased SOD and increased MDA in serum. Moreover, XJEK treatment also improved endothelial dysfunction (ED) with not only enhanced eNOS activities in serum and cardiac tissues and elevated NO levels in serum, but also decreased Ang II content in serum and cardiac tissues. Finally, protein expressions of pro-inflammation cytokines, TNF-α and IL-1β in the cardiac tissues with XJEK treatment were significantly decreased compared to model. On the contrary, IL-10, an anti-inflammatory cytokine concentrated in cardiac tissues was significantly enhanced compared to model.

**Conclusion:**

Xinji′erkang exerts cardioprotective effect on myocardial infarction in mice, which may be due to the improvement of endothelial dysfunction and the reduction of oxidative stress and inflammation response.

**Electronic supplementary material:**

The online version of this article (doi:10.1186/s12906-017-1846-5) contains supplementary material, which is available to authorized users.

## Background

Myocardial infarction (MI) is one of the prominent risk factors responsible for morbidity and mortality as a major public health hazard [1]. Recent findings have demonstrated the important roles of cardiac hypertrophy, fibrosis and apoptosis in myocardial injury [2, 3]. Heart failure and sudden cardiac death are ultimately developed due to over-loading of the heart, which resulting in impaired cardiac function [4, 5]. For the prevention of the progression of heart failure, it is critical to block or at least slowdown the cardiac pathological changes. Pathological remodeling of left ventricular (LV) is commonly associated with MI [6]. Structural changes of the myocardium including intracellular and extracellular LV wall, and LV dilation in response to pressure over-load may happen as the consequences of the LV remodeling. Thus, condition of LV remodeling could be used as a predictive factor for mortality and morbidity in patients with MI [7]. Electrocardiography (ECG) has become the most commonly used technique for the assessment of heart disease [8]. Wang et al. reported that the use of the ECG score system was the best diagnositic tool in the estimation of ischemia conditions with quantitative analysis of change in the ST-segments for either anterior or inferior LV [9]. Despite significant progress in recently FDA approved drugs, such as inhibitors of angiotensin converting enzyme, blockers of an adrenergic receptor and angiotensin II type 1 receptor (AT_1_R), MI remains a deadly clinical problem [10].

Therefore, there is a crying need to develop unprecedented and therapeutically efficient medicines for MI. The application of alternative medicines for cardiovascular diseases has increased rapidly [10]. Xinji′erkang (XJEK) is a Chinese herbal formula, which consists of fourteen herbal medicines, including *Panaxginseng,C.A.Mey., Astragalusmongholicus Bunge, Ophiopogonjaponicus (Thunb.)KerGawl, Polygonatumodoratum (Mill.) Druce*etc [11].It has been reported that its therapy clinically improved cardiac function with evidences showing its curative effects on several heart diseases such as coronary heart disease, viral myocarditis and toxic myocarditis [12]. Animal studies proved as well its protective effects against ventricular hypertrophy of isoproterenol-induced mice. In 2K1C rats, it also exerted protective effects on cardiac remodeling [13]. In addition, our previous studies demonstrated that the protective effects of XJEK against isoproterenol-induced ventricular remodeling were due to its actions in decreasing the oxidative stress (OS) and increasing the antioxidative activities [14].

However, whether XJEK improves MI outcomes in mice remains unrevealed. The focus on the current study is to test whether the chronic administration of XJEK reverses MI-induced cardiovascular remodeling in mice, and to explore the underlying mechanisms of its role in ED, OS and inflammation pathways. Hopefully, the results in this study may lay a foundation for its clinical application in the treatment of MI.

## Methods

### Experimental animals

Forty male KM mice (weighed 28–30 g), aged eight weeks, purchased from Shanghai Slac laboratory animal Corp. Ltd. [Certificate No.SCXK (HU) 2012–0002] were used in this study and cared in animal house at Anhui Medical University. All experimental procedures were approved by the Committee on the Ethics of Animal Experiments of Anhui Medical University. Mice were kept in groups of 3–5 per cage and maintained lights on at 6 am for providing 12-h light-dark cycle. The room of mice was maintained at 25 °C as appropriate temperature. During the four weeks, all experiment mice had access to water and food ad libitum.

### Chemicals

XJEK was obtained from Hefei Company of Traditional Crude Drugs (Hefei, China), which consists of 14 different medicinal herbs (As shown in Table 1). The preparation of XJEK aqueous extract and the HPLC fingerprint have been reported by our group previously [11]. Fosinopril was purchased from Bristol-Myers Squibb (Shanghai, China). Cefoxitin sodium was bought from *Li jian medicine* (Shenzhen, China).

**Table 1:** Recipe of XJEK formulation

Components	Voucher specimens number	Part used	Rate (%)
*Panax ginseng* C.A. Mey.	PCAHMU-20121005	Root	11.71
Polygonatum odoratum (Mill.) Druce	PCAHMU-20121006	Rhizome	7.03
Panax pseudo ginseng var. No to ginseng (Burkill) G. Hoo & C.L. Tseng	PCAHMU-20121007	Root	3.09
Allium macrostemon Bunge	PCAHMU-20121008	Ramulus	7.80
Angelica sinensis (Oliv.) Diels	PCAHMU-20121009	Root	7.80
*Ophiopogon japonicus* (Thunb.) Ker Gawl.	PCAHMU-20121010	Root	7.80
Schisandra chinensis (Turcz.) Baill.	PCAHMU-20121011	Fruit	3.93
Salvia miltiorrhiza *F. alba* C.Y. Wu & H.W. Li	PCAHMU-20121012	Root	7.80
*Sophora flavescens* Aiton	PCAHMU-20121013	Root	7.80
Glycyrrhiza acanthocarpa (Lindl.) J.M. Black	PCAHMU-20121014	Rhizome	7.80
*Astragalus mongholicus* Bunge	PCAHMU-20121015	Root	11.69
Epimedium acuminatum Franch.	PCAHMU-20121016	Aerial part	7.80
Trichosanthes obtusiloba C.Y. Wu	PCAHMU-20121017	Seed	7.80
*Dryobalanops aromatica* C.F. Gaertn.	PCAHMU-20121018	Resin	0.15

### Stereotaxic surgery procedures of MI

As previously reported, stereotaxic surgery MI procedures were performed [15, 16]. Briefly, mice were anesthetized with pentobarbital sodium (45 mg/kg, intraperitoneal injection). Subsequently, the thoracic cavity was opened and the heart was exposed in surgery. A 7–0 sterile surgical suture was used to ligature the left anterior descending (LAD) coronary artery for subjected to occlude bloodstream. In sham mice, the suture was only placed around the artery without ligation. The chest was closed with a 3–0 sterile surgical suture after positive end-diastolic pressure was applied to fully inflate the lung, and wounds were cleaned and disinfected. Successful MI was not only notarized by the appearance of regional epicardial cyanosis over the myocardial surface and myocardial infarction performance of ECG (elevation of the ST segment or high and pointed T wave), but also by assessment of infarct size with triphenyltetrazolium chloride (TTC) stained (see Additional file 1: Fig. S1). During the recovery periods of the following surgery operation, mice were given cefoxitin sodium (200 mg/kg/day) for three consecutive days.

### Drug administration in four different groups

Forty mice were randomly assigned into four groups as follows (*n* = 10 per group): Sham group (without LAD occlusion and only received an intragastric gavage of 10 ml/kg/day distilled water), model group (ligation of LAD and received an intragastric gavage of 10 ml/kg/day distilled water), XJEK group(ligation of LAD and received an intragastric gavage of XJEK at the dose of 7.5 g/kg/day), and fosinopril group (ligation of LAD and received an intragastric gavage of fosinopril at the dose of 20 mg/kg/day), and all groups were administered accordingly for four weeks.

### Cardiac function studies by electrocardiogram

At the end of the 4th week according to the date of euthanasia, the animals were anaesthetized with pentobarbital sodium (45 mg/kg, intraperitoneal injection), and then were set on the operating table in supine position. In accordance with the principle of electrocardiogram, four electrodes are inserted into the subcutaneous tissue of the mice limbs, and then the signals were recorded on a four channel direct-writing oscillograph (BL420S, Chengdu Techman Software Co.,Ltd., China), digitally sampled system (1 kHz) on a personal computer equipped with an analogue to digital interface, the height of P and S wave, the width of P wave QRS wave and T wave were measured, as well as the time of P-R and Q-T (BL-420S biological data acquisition and analysis system, Chengdu Techman Software Co., Ltd., China).

### Hemodynamics and cardiac remodeling index

After ECG recording, the cardiac index was assessed by hemodynamics, the right carotid artery was cannulated with a polyethylene catheter connected to a Statham, and the mean carotid artery pressure was measured. Then the catheter was inserted along the right coronary artery into the left ventricle, and the signals were recorded on a four channel direct-writing oscillograph (BL420S, Chengdu Taimeng Software Co. Ltd.). A digitally sampled (1 kHz) system is a personal computer equipped with an analogue-to-digital interface (biological function experiment system, BL420S). The heart rate (HR), the artery systolic blood pressure (ASBP), the left ventricular systolic (LVSP) and end-diastolic pressures (LVEDP), the maximal rate of left ventricular systolic and diastolic pressure (±dp/dtmax) were recorded by a computer-based automatic real-time detection system for the hemodynamic parameters of cardiovascular system (Transonic Scisense Inc., U.S.A).

### Serum and tissue collection

Thereafter, blood was collected from the left carotid artery in the tubes and was immediately centrifuged with 3500 rpm for 10 min at 4 °C, and the serum was stored at −80 °C until being analyzed. Then animals were sacrificed by exsanguination. The thoracic cavity was opened for collecting hearts and aortas. Hearts were washed in ice-cold saline solution, blotted, photographed and weighed. The heart weight/body weight (HW/BW) was calculated, then the heart samples were sliced into several parts for various analysis and were kept in −80 °C for further studies.

### Histological examination

After weighed, the right amount of the apex in neutral 10% buffered formalin was cut for histological analysis. From each paraffin block, four slices (5um) were stained with hematoxylin and eosin (HE) and Van Gieson (VG) according to the standard protocol. The myocyte cross-sectional area (CSA), the collagen volume fraction (CVF) and perivascular circumferential collagen area (PVCA) from HE and VG staining, respectively, were quantitatively analyzed with Image J (1.61) in digitalized microscopic images as have been previously described. Thoracic aorta was removed from mice and maintained in neutral 10% buffered formalin. Paraffin-embedded thoracic aorta (5um) was cut, dewaxed and stained with HE. The structural changes of aorta were investigated using a light microscope as has been previously reported. The CSA, aorta radius (AR), total aorta area (TAA) and media thickness (MT) of aorta were recorded and focused on quantifying about the effect of aorta change.

### Measurements of NO, SOD and MDA in serum

Following the method described by Veltkamp et al. (2002), Nitric oxide (NO) content was determined, and most of NO underwent a quick change to nitrite (NO^2−^) and dinitrate (NO^3−^) due to its unstability in physiological solutions. The serum levels of NO^2−^/NO^3−^were determined using NO detection Kit based on the manufacturer’s instructions. In brief, nitrate was converted to nitrite under the influence of aspergillus nitrite reductase, and the total nitrite was quantified by the Griess reagent. A spectrophotometer at 540 nm was applied to measure absorbance. The malondialdehyde (MDA) content was quantified by the thiobarbituric acid reactive substances (TBARS) assay on the basis of the manufacturer’s instructions (Jiancheng Institute of Bioengineering Company, Nanjing, China) and the absorbance was determined at a wave length of 532 nm. Superoxide dismutase (SOD) activity was observed by determining the absorbance at 550 nm with the use of SOD assay kit (Jiancheng Bioengineering, Nanjing, China).

### Measurements of serum endothelial NO synthetase (eNOS) and Ang II in serum and cardiac tissues

Conforming to the manufacturer’s instructions, the content of serum eNOS was tested by the enzyme linked immunosorbent assay (ELISA) and Ang II in serum and cardiac tissues were determined by using the ELISA kits as well.

### Measurement of pro-inflammatory cytokines (TNF-α and IL-1β) and anti-inflammatory cytokine (IL −10) in cardiac tissues

The protein levels of pro-inflammatory cytokines (TNF-α and IL-1β) and anti-inflammatory cytokine (IL-10) in cardiac tissues were determined by using commercially available ELISA kits (Company name). Briefly, 0.1 g of frozen tissues were added with 1 ml lysate buffer, containing 1 mol/l phenylmethylsulfonyl fluoride, 1 mg/l pepstatin A,1 mg/l aprotinin and 1 mg/l leupeptin in PBS (pH = 7.2), homogenized by a glass homogenizer and centrifuged with 3000 rpm for 10 min at 4 °C. Protein concentration was determined by Bio-Rads DC™ Protein Assay kit. 1 μg samples were used to determine IL-1β, TNF-α, and IL-10 protein levels by ELISA kits. The procedure was fulfilled according to manufacturer’s specification via the application of the mice-specific ELISA kits, and the color reaction was detected using the chromogen tetramethyl benzidine. Color reaction was blocked by an equal volume of stop solution (provided by the manufacturer) and processed by a microplate reader at a wavelength of 450 nm, the color change was in proportion to the concentration of the cytokines measured, and all samples were within the range of the standard curve.

### Immunohistochemistry of eNOS

Immunohistochemical staining was carried out with the utilization of UltraSensitive S-P kit manufactured by Boster-Bio (China). According to the standard protocol, sections were deparaffinized and microwave-treated for 10 mins twice in 10 mM sodium citrate (ph 6.0). The endogenous peroxidase was blocked in endogenous peroxidase blocking solution by a10-minute incubation in the room temperature. Rabbit polyclonal antibodies against eNOS were employed as primary antibodies for 18 h in a 1:100 dilution at 4 °C. The sections were washed three times with phosphate-buffered saline (PBS), and then incubated with a secondary antibody for 10 mins. They were washed with PBS again before treated with streptoavidin-perooxidase for 10 mins. Ultimately, specimens were incubated in diaminobenzidine for 5 mins followed by haematoxylin counterstaining. The images were recorded by digital camera system (Leica DM IL, DC 300), and then eNOs were quantitatively analyzed with Image J (1.61) in digitalized microscopic images.

### Statistical analysis

The experimental data were expressed as mean ± SEM, and data were evaluated using the statistical analysis program (GraphPad prism 6). Statistics were performed with one-way analysis of variance (ANOVA).The *P* value of less than 0.05 was considered significant statistically.

## Results

During the period of four weeks, ten mice died of surgery-induced injury due to inflammation or infection. Thus, at the end of four weeks, the data of sham group (*n* = 9), model group (*n* = 6), XJEK group (*n* = 8) and fosinopril group (*n* = 7) were evaluated respectively. Four weeks after MI, the echocardiography and hemodynamics were measured as the data of cardiac function of MI mice, which were then anesthetized for the collection of blood for serum NO, eNOS, Ang II, SOD and MDA measurements and the harvest of hearts for histological analysis, as well as the measurement of AngII, pro-inflammatory cytokines and anti-inflammatory cytokines in cardiac tissues.

### Cardiac function assessment by ECG analysis

**Fig. 1:**
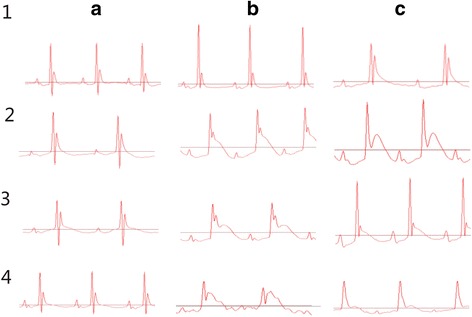
Representative figures of ECG in different groups of mice. **a** Representative figure of pre-MI; **b** Representative figure of post-MI; **c** Representative figure of 4 wks after MI. (1) Sham group; (2) Model group; (3) XJEK group; (4) Fosinopril group

Cardiac function was assessed by ECG and representative images before, after MI with or without drug administration was shown in Fig. 1 and Table 2. Mice in different groups presented similar electrocardiogram before surgery. After surgery-induced MI, mice with MI had significant changes in electrocardiogram compared to those of sham group (*P* < 0.05). Four weeks after XJEK medication in mice, a significant improvement emerged on ECG as following: the height of P wave was raised and width of P wave was declined compared to that of sham group (*P* < 0.05); the width of QRS, S and T wave significantly decreased compared to that of sham group (*P* < 0.05) and the time of P-R and Q-T also decreased markedly compared to that of sham group (*P* < 0.05). Similar results were displayed in fosinopril treatment group.

**Table 2:** Effects of XJEK on electrocardiogram remodeling of MI mice (mean ± SEM)

Group	*n*	Height of P(mv)	Width of P(ms)	Width of QRS(ms)	Time of P-R(ms)	Time of Q-T(ms)	Width of T(ms)	Height of S(mv)
Sham	9	0.11 ± 0.01	13.67 ± 0.53	23.56 ± 1.74	31.11 ± 2.34	27.33 ± 0.65	7.44 ± 0.56	−0.20 ± 0.02
Model	6	0.08 ± 0.01^*^	17.00 ± 0.58^**^	30.17 ± 2.80^*^	38.33 ± 2.03^*^	33.17 ± 3.09^*^	9.83 ± 1.01^*^	0.11 ± 0.01^**^
XJEK	8	0.12 ± 0.01^#^	12.25 ± 1.15^##^	21.75 ± 1.50^##^	25.50 ± 2.92^##^	26.29 ± 1.20^#^	7.13 ± 0.44^#^	−0.19 ± 0.02^#^
Fosinopril	7	0.11 ± 0.01^#^	13.14 ± 1.08^##^	21.71 ± 1.63^#^	23.71 ± 1.41^##^	24.50 ± 2.00^#^	7.14 ± 0.63^#^	−0.17 ± 0.01^##^

^*^ *P* < 0.05, ^**^ *P* < 0.01 vs. Sham group; ^#^ *P* < 0.05, ^#^ *P* < 0.01 vs. Model group

### Hemodynamics assessment

As was described in Table 3, HR of MI mice increased significantly compared to that of sham group (*P* < 0.01). However, no change was determined in ASBP, LVSP, LVEDP and ±dp/dt_max_ between model group and sham group, indicating that the cardiac function of MI mice was in compensatory stage and proceeded to discompensatory stage (see Additional file 2: Fig. S2). Four weeks after administration of XJEK and fosinopril, HR in both groups decreased obviously compared to that in sham group(*P* < 0.01 and *P* < 0.05), and ±dp/dt_max_ also markedly decreased compared to that of model group (*P* < 0.01), which indicated that XJEK may preserve and prolong the compensatory reaction, with similar efficacy to that of fosinopril treatment.

**Table 3:** Effects of XJEK on cardiac function of MI mice (mean ± SEM)

Group	*n*	HR(Times/min)	ASBP(mmHg)	LVSP(mmHg)	LVEDP(mmHg)	dp/dtmax(mmHg/s)	-dp/dtmax(mmHg/s)
Sham	9	413.75 ± 6.03	90.36 ± 2.66	84.58 ± 2.91	−1.97 ± 1.17	4665.30 ± 107.62	−3792.47 ± 114.30
Model	6	456.60 ± 3.82^**^	90.79 ± 6.13	89.67 ± 5.39	−1.62 ± 0.87	4816.32 ± 158.86	−4162.92 ± 137.04
XJEK	8	376.86 ± 16.22^##^	84.98 ± 8.87	85.35 ± 3.75	−0.47 ± 0.83	3786.10 ± 213.42^##^	−2977.76 ± 153.14^##^
Fosinopril	7	427.50 ± 11.08^#^	78.66 ± 8.12	83.55 ± 7.32	−1.13 ± 3.34	3551.12 ± 296.28^##^	−2818.68 ± 281.20^##^

^*^ *P* < 0.05, ^**^ *P* < 0.01 vs. Sham group; ^*#*^ *P* < 0.05, ^*##*^ *P* < 0.01 vs. Model group

### Cardiac structure remodeling studies

**Fig. 2:**
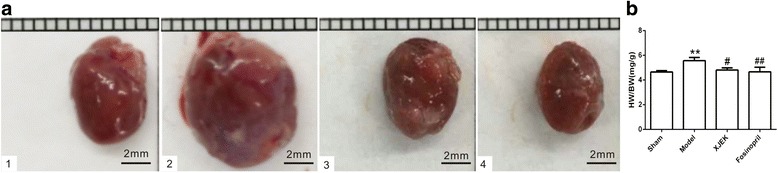
Effect of XJEK on HW/BW of MI mice. **a** Representative figure of heart macroscopic images; **b** Statistic results (mean ± SEM, *n* = 6–9). (1)Sham group; (2) Model group; (3) XJEK group; (4) Fosinopril group. ^*^ *P* < 0.05, ^**^ *P* < 0.01 vs.Sham group; ^*#*^ *P* < 0.05, ^*##*^ *P* < 0.01 vs.Model group

**Fig. 3:**
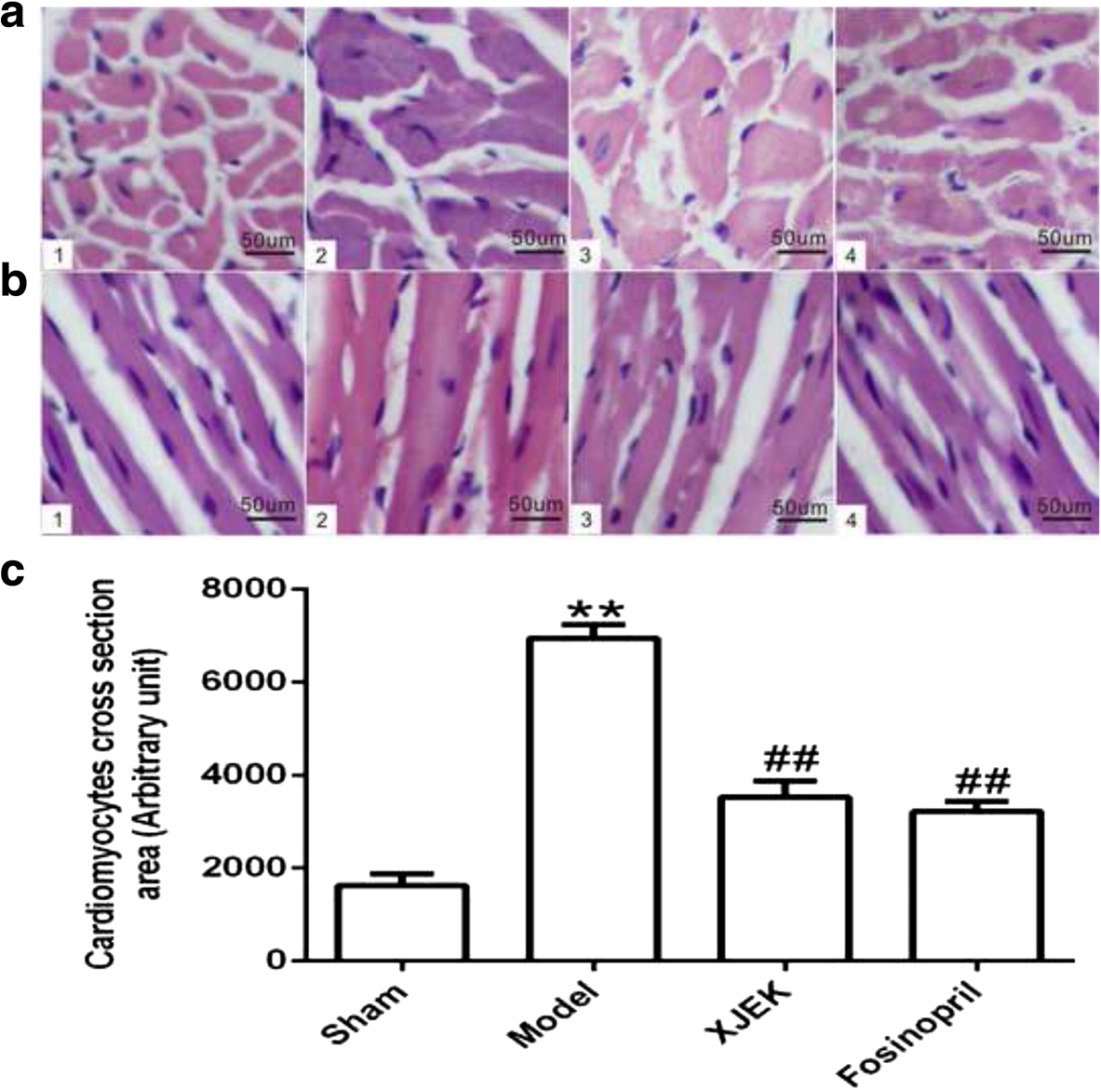
Effect of XJEK on cardiomyocyte CSA and cardiomyocyte long axis of MI mice (HE stain, magnification × 400). **a** Representative images of histological section of cardiomyocyte cross-section (HE staining, magnification × 400); **b** Representative images of histological section of cardiomyocyte long axis (HE staining, magnification × 400); **c** Quantitative analyses results (mean ± SEM, *n* = 6–9). (1)Sham group; (2) Model group; (3) XJEK group; (4) Fosinopril group. ^*^ *P* < 0.05, ^**^ *P* < 0.01 vs. Sham group; ^*#*^ *P* < 0.05, ^*##*^ *P* < 0.01 vs. Model group

**Fig. 4:**
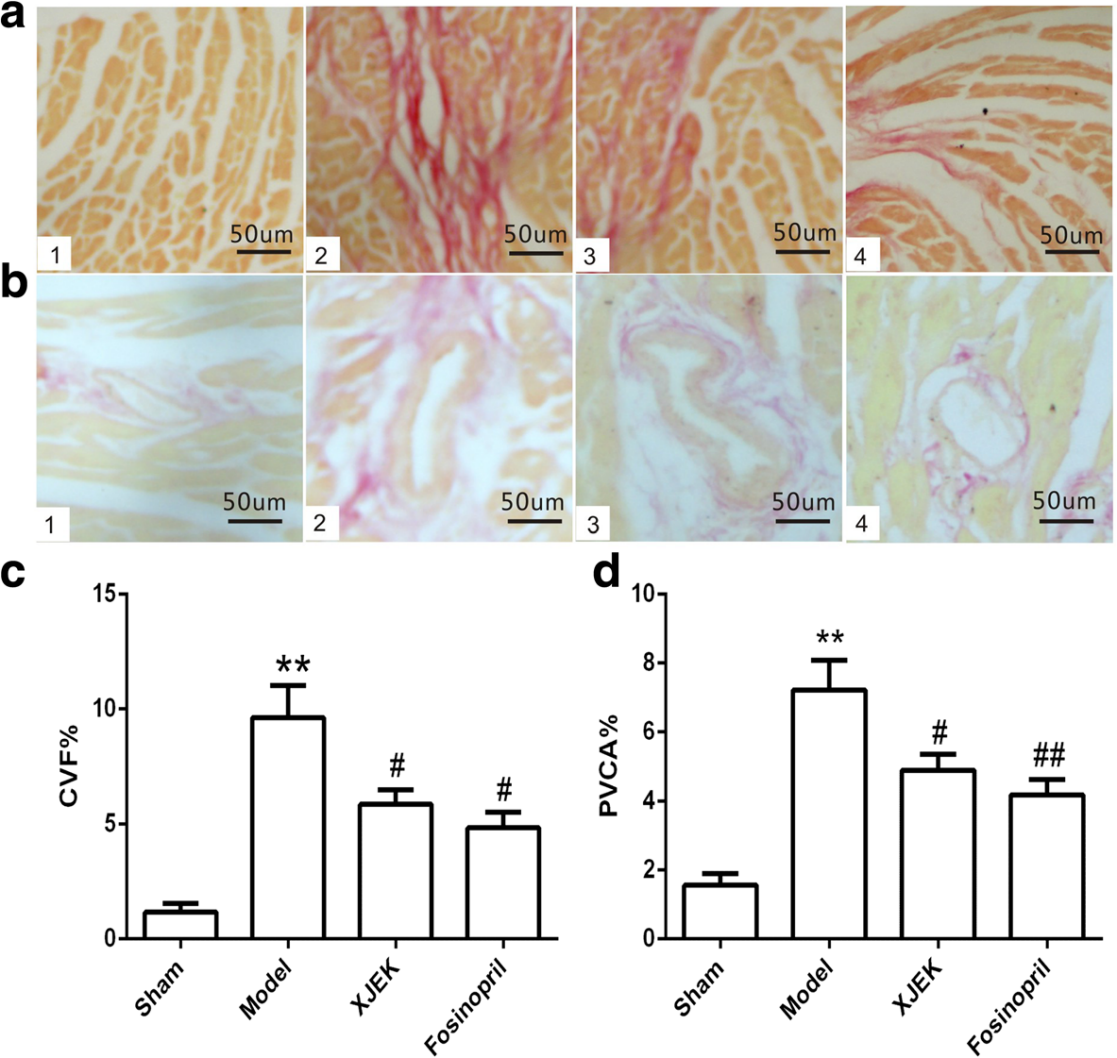
Effect of XJEK on cardiac tissue PVCA and CVF of MI mice (VG staining, magnification ×400). **a** Representative images of histological section of CVF (VG staining, magnification ×400); **b** Representative images of histological section of PVCA (VG staining, magnification × 400); **c** Quantitative analyses of CVF (mean ± SEM, *n* = 6–9); **d** Quantitative analyses of PVCA (mean ± SEM, *n* = 6–9); (1) Sham group; (2)Model group; (3) XJEK group; (4) Fosinopril group. ^*^ *P* < 0.05, ^**^ *P* < 0.01 vs. Sham group; ^*#*^ *P* < 0.05, ^*##*^ *P* < 0.01 vs. Model group

For the quantitative analysis of cardiomyocyte hypertrophy, HW/BW ratio was revealed in Fig. 2, and it was apparent that HW/BW ratio of model group significantly increased compared to that of sham group(*P* < 0.01). Additionally, HW/BW index in mice with XJEK treatment and fosinopril treatment was significantly reduced compared to that of sham group (*P* < 0.01). Cardiomyocyte CSA, the levels of CVF and PVCA of model group strikingly increased compared with those of sham group (*P* < 0.01, Fig. 3a-c and Fig. 4a–d). Those pathological changes were attenuated significantly in mice with XJEK and fosinopril administration.

### Aortic remodeling studies

**Fig. 5:**
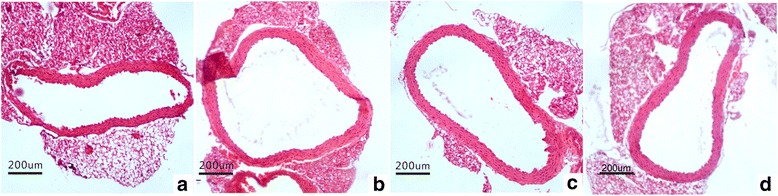
Representative images of thoracic aorta remodeling in different groups of mice (HE staining, magnification ×200). (**a**)Sham group; (**b**) Model group; (**c**) XJEK group; (**d**) Fosinopril group

The vascular remodeling of the upper thoracic aorta exposed to MI was observed at the end of four weeks. When compared to sham group, the values of the area of the TAA, CSA, AR and MT of the aorta in MI mice significantly increased (*P* < 0.01), which then could be blocked by the treatment with XJEK and fosinopril (Fig. 5 and Table 4).

**Table 4:** Effects of XJEK on thoracic aorta remodeling of MI mice (mean ± SEM)

Group	*n*	TAA(10^3^um^2^)	LA(10^3^um^2^)	CSA(10^3^um^2^)	AR(um)	Lumen(um)	Media(um)	Media/ lm (%)
Sham	9	116.04 ± 6.03	102.79 ± 5.60	13.25 ± 1.84	6.07 ± 0.16	5.71 ± 0.16	0.36 ± 0.05	6.30 ± 0.81
Model	6	190.82 ± 10.98^**^	156.51 ± 8.99^**^	34.31 ± 2.97^**^	7.78 ± 0.22^**^	7.05 ± 0.20^**^	0.73 ± 0.05^**^	10.43 ± 0.74^**^
XJEK	8	141.25 ± 9.08^##^	120.10 ± 8.70^#^	21.16 ± 1.17^##^	6.70 ± 0.22^##^	6.17 ± 0.23^##^	0.53 ± 0.03^#^	8.60 ± 0.71
Fosinopril	7	132.34 ± 6.86^##^	113.73 ± 5.92^##^	18.90 ± 1.45^##^	6.50 ± 0.17^##^	6.01 ± 0.16^##^	0..48 ± 0.03^#^	7.99 ± 0.48^#^

^*^ *P* < 0.05, ^**^ *P* < 0.01 *vs.* Sham group; ^*#*^ *P* < 0.05, ^*##*^ *P* < 0.01 *vs.* Model group

### Serum NO, SOD and MDA assessments

**Fig. 6:**
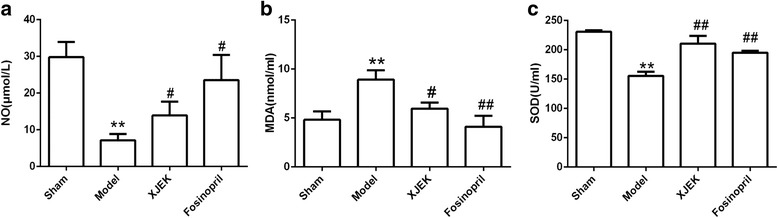
Effect of XJEK on NO content (**a**), MDA content (**b**) and SOD content (**c**) in serum of MI mice (mean ± SEM, *n* = 6–9). ^*^ *P* < 0.05, ^**^ *P* < 0.01 vs. Sham group; ^*#*^ *P* < 0.05, ^*##*^ *P* < 0.01 vs. Model group

The concentrations of NO and SOD were markedly reduced in model group compared with those in sham group (*P* < 0.01). Interestingly, XJEK treatment reversed MI-induced decrease of NO and SOD (*P* < 0.05, Fig. 6a and c). In contrast, serum MDA significantly increased in model group compared to that in sham group (Fig. 6b, *P*<0.01).Whereas, XJEK and fosinopril treatment brought reduction in MDA level (*P* < 0.05).

### Measurement of Ang II content in serum and cardiac tissues

**Fig. 7:**
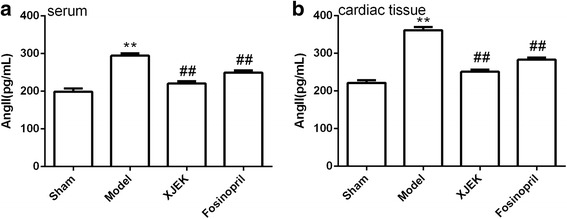
Effect of XJEK on Ang II content in serum (**a**) and cardiac tissues (**b**) of MI mice (mean ± SEM, *n* = 6–9). ^*^ *P* < 0.05, ^**^ *P* < 0.01 vs. Sham group; ^*#*^ *P* < 0.05, ^*##*^ *P* < 0.01 vs. Model group

As shown in Fig. 7, compared with sham group, MI mice displayed high levels of Ang II in serum and cardiac tissues (*P* < 0.01). Treatment with XJEK and fosinopril for four weeks could markedly inhibit the increase of Ang II in serum and cardiac tissues.

### Assessment of eNOS concentration in serum and eNOS expression in cardiac tissues

**Fig. 8:**
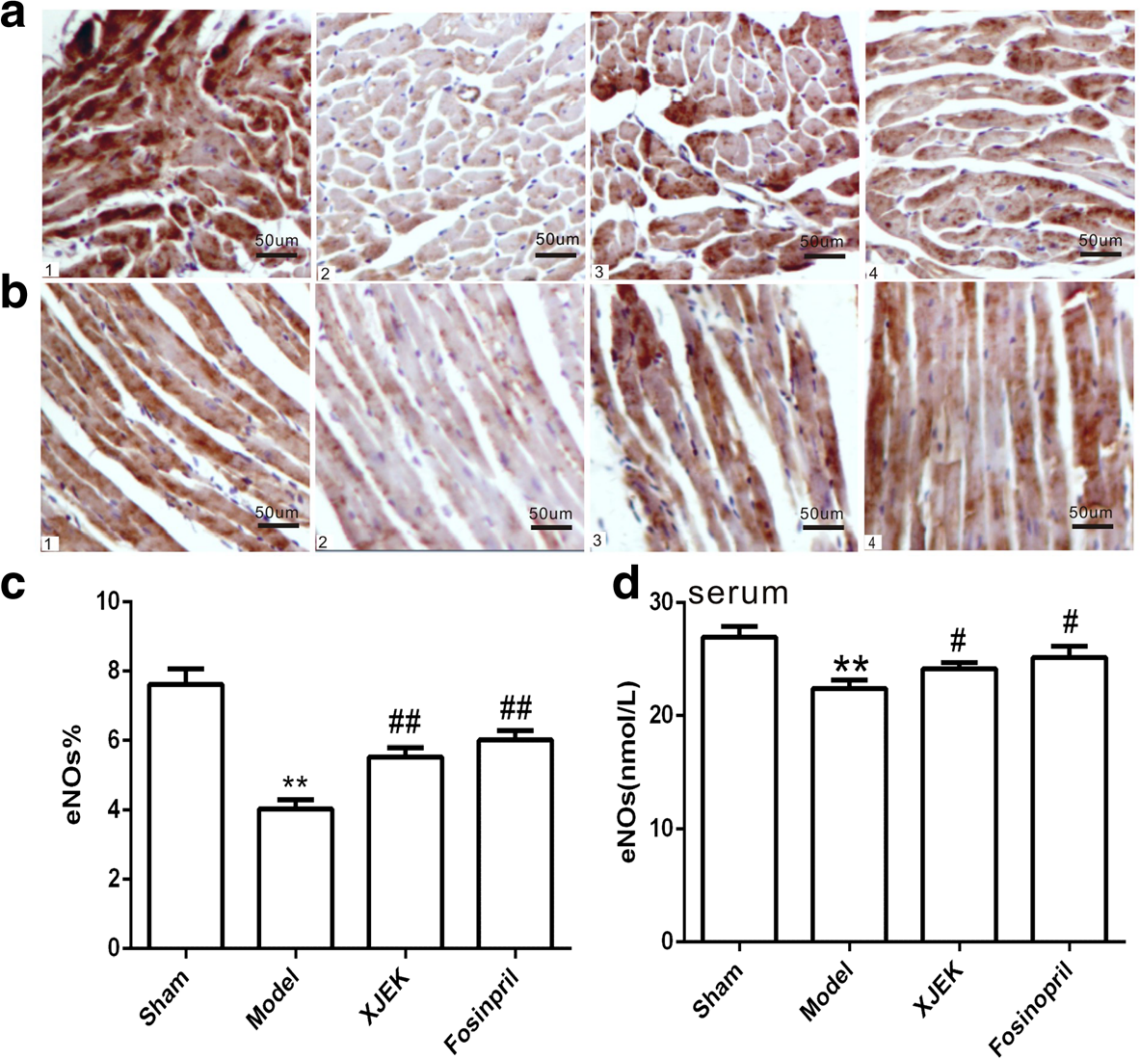
Effect of XJEK on eNOS content in serum and cardiac tissues of MI mice. **a** Representative images of cardiomyocyte cross-section with immunohistochemistry (magnification × 400); **b** Representative images of cardiomyocyte long axis with immunohistochemistry (magnification × 400); **c** Quantitative analyses results (mean ± SEM, *n* = 6–9). **d** eNOS content in serum (mean ± SEM, *n* = 6–9) (1) Sham group; (2) Model group; (3) XJEK group; (4) Fosinopril group. ^*^ *P* < 0.05, ^**^ *P* < 0.01 vs. Sham group; ^*#*^ *P* < 0.05, ^*##*^ *P* < 0.01vs.Model group

eNOS in serum was markedly reduced in model group compared with that in sham group (*P* < 0.01). Interestingly, XJEK treatment reversed MI-induced decrease of serum eNOS (*P* < 0.05, Fig. 8d). As shown in Fig. 8 by immunohistochemical detection, eNOS protein expression in cardiac tissues showed a down-regulation in model group compared to that in sham group (*P* < 0.01), but treatment with XJEK significantly up-regulated the expression of eNOS, just as the treatment with fosinopril (*P* < 0.01, Fig. 8a-c).

### Measurement of TNF-α and IL-1β, and IL-10 in cardiac tissues

**Fig. 9:**
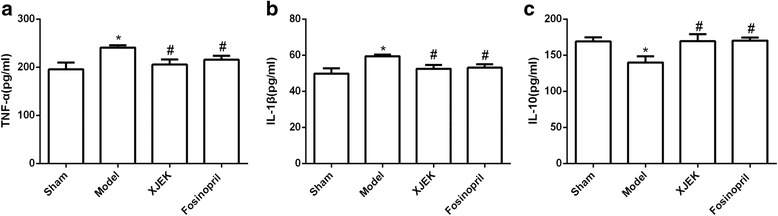
Effect of XJEK on TNF-1α (**a**), IL-1β (**b**) and IL-10 (**c**) content in cardiac tissues of MI mice (mean ± SEM, *n* = 6–9). ^*^ *P* < 0.05, ^**^ *P* < 0.01 vs. Sham group; ^*#*^ *P* < 0.05, ^*##*^ *P* < 0.01vs.Model group

We examined the protein expression of pro-inflammatory cytokines (TNF-α and IL-1β) as well as anti-inflammatory cytokines (IL-10) by ELISA kits. As exhibited in Fig. 9, TNF-α and IL-1β were markedly elevated in model group compared to those in sham group (*P*<0.05). In contrast, four weeks after administration of XJEK after MI, the protein expression of IL-1β and TNF-α was significantly decreased in cardiac tissues compared to model group, and the similar results were observed in mice with fosinopril treatment. In addition, we also assayed the protein expression of IL-10 and hereby found that IL-10 level was persistently down-regulated in model group. The protein expression of IL-10 in mice with XJEK and fosinopril administration after MI was significantly increased compared to that of model group (*P* < 0.05).

## Discussion

MI, which is usually induced by myocardial ischemia, often brings about the decrease of cardiac oxygen-rich blood flow. MI may promote the thinning of infarcted area of remote myocardium, resulting in progressive enlargement of the LV wall [17]. LV chamber changes from ellipsoidal to spherical shape, with reduction in long to short axis ratio [18]. Several biological processes are involved in the time course of remodeling after MI: i local ischemia and myocardial cell death; ii oxidative stress and inflammatory reactions in injured myocardium and subsequent cardio-depressive reactions [19, 20]; iii increased synthesis of collagens and myocardial fibrosis following the activation of matrix metalloproteinase (MMPs) [21, 22]; vi response of mechanical stress to changes in myocardial intra- and extracellular structures [23]. Increased HW/BW ratio and myocyte CSA, with the extent of collagen deposition in the cardiac interstitium, and the amount of collagen deposition in the cardiac interstitium are often associated with the development of acute to chronic stage of MI. Untreated MI induces progressive LV dysfunction [24]. The current research demonstrates that oral administration of XJEK exerts its beneficial effects on cardiovascular remodeling [25]. An established drug was thus used in the treatment of cardiovascular remodeling of MI mice with attenuation of progressive heart dysfunction, evidenced by normalized LVEDP, ±dp/dt_max_ and cardiac hypertrophy, and reduced HW/BW ratio and cardiomyocyte CSA, improved reactive interstitial fibrosis in the cardiac interstitium and vascular remodeling.

The specific change of the electrocardiogram in MI described in numerous researches is the reduction of repolarizing potassium currents in the infarct border zone, which extends action potential duration (APD) and mishandles intracellular calcium, leading to arrhythmia [26]. ECG is an essential tool frequently applied in the detection of cardiac electrical signals that represent heart activity potentials. ST-segment elevation is a hallmark feature for MI [27]. In MI patients, the QRS score about ECG has been used as an indicator of the severity of cardiac damage [28]. Taken together, quantitative assessments based on ECG scoring system in estimation of the extent of ischemic conditions are of great use for evaluating the extent and prognosis of acute ischemia, taking account of ST-segment and QRS score [29]. Interestingly, XJEK reduces QRS, P-R, Q-T interval, P wave and T wave width, and increases the ST-segment and P wave height compared to MI group, indicating that XJEK exerts protective effects on MI.

ED was reported to occur in acute cardiovascular diseases such as MI. NO is synthesized from *L*-arginine by the enzyme, and NOS is an important mediator regulating endothelial functions [30]. NO is potent ventricular dilator which blocks the progression of MI [31]. Increasing studies in recent years have confirmed the critical role of NO formation in the arteries in response to cholinomimetic stimulation in MI mice, which reduces the reactive oxygen species (ROS) production and enhances eNOS activities [32]. Mice with deletion of eNOS enzyme suffer spontaneous MI [33]. Remarkably, mice lacking in triple NOS enzymes also developed spontaneous MI, and 55% of mice died of MI eventually [34]. In the current study, serum NO level and eNOS activity were impaired in MI group, which was then restored four weeks post the administration of XJEK, similar to those in fosinopril group. Ang II, the main effector molecule of RAS, produces superoxide anion via Ang II type 1 receptor (AT_1_R) and activation of the reduced form of nicotinamide adenine dinucleotide phosphate (NADPH) oxidase. Experimental and clinical studies have consistently confirmed that MI is associated with activation of the systemic RAS with increased concentration of angiotensin peptides in the blood and changes in expression of angiotensin receptors [35]. The harmful effects of Ang II via AT_1_R induce not only hypertension but also inflammatory, hypertrophic and fibrotic reactions [36]. These effects of Ang II are correlated with the pathophysiology of MI. MI induces morphological changes called remodeling, which leads to heart failure accompanied by infarct area extension and thinning and compensatory hypertrophy of the non-infarcted myocardium [37]. Ang II-induced reactions may further damage the myocardium and accelerate post-MI remodeling [38]. Our present study demonstrated that Ang II in serum and cardiac tissues was markedly up-regulated in model group and then decreased strikingly after treatment with XJEK and fosinopril for four weeks in mice with MI.

OS in cardiovascular system plays an important role in cardiovascular diseases, in terms of its aggravation of cardiomyocyte hypertrophy and myocardium failure. Several antioxidants in mice play its role in ameliorating heart failure [39]. Consistent with current research, we also find that OS is involved in MI induced cardiovascular remodeling. Detrimental effects of ROS are clearly demonstrated in MI mice, with an apparent decline in an antioxidant protein SOD and overexpression of MDA [40, 41]. OS-induced activation of transcription factors leads to synthesis of antioxidant enzymes such as manganese SOD and eNOS. Thus, ROS is a key player for cell injury in response to mechanical stress such as OS. Increased lipid peroxides in MI mice accumulate lipids in heart and blood vessels, which eventually brings about the irreversible damages in the target organs. Under the pathologic conditions, excessive reactive oxygen species resulted in the imbalance of antioxidant system. MDA, the end product of lipid pero-xidation, was overproduced in the MI mice. SOD is considered as the dominant enzymes functioning as free radical scavengers, the vitality of which is dramatically suppressed [42]. In agreement with these previous literatures, the model group displayed the same OS status, in addition, the levels of MDA was down-regulated with XJEK treatment for 4 weeks and the activities of SOD was recovered versus those in MI mice, as well as fosinopril.

Inflammation is commonly associated with MI as increased levels of pro-inflammatory cytokines [43]. In this study, it was detected that the level pro-inflammatory cytokines including TNF-α and IL-1β increased, whereas anti-inflammatory cytokine, IL-10, decreased in MI. TNF-α is produced by a variety of cells, including multiple cells of the immune system such as fibroblasts, endothelial cells and neuronal cells. It down-regulates endothelial NO production, inducing hypertension thereafter. Additionally, it also adjusts eNOS gene expression at transcriptional level by suppressing the activity of promoter, resulting in decreased eNOS protein expression and reduced NO in the endothelium [44]. Recent studies have also revealed that microRNA155 is involved in the regulation of eNOS at mRNA level and reducing endothelium-dependent vasodilatation in response to TNF-α [45]. Elevated levels of another pro-inflammatory cytokine, IL-1β, have been linked to the increased morbidity and mortality in MI. The pro-inflammatory cytokine activation in the myocardium leads to cardiomyocyte apoptosis in MI [46]. These findings are in agreement with current studies in terms of the role of these pro-inflammatory cytokines in model of MI. IL-10, a multifunctional anti-inflammatory cytokine that down-regulates cell-mediated immune responses and cytotoxic inflammatory responses [47], is primarily considered due to its capacity to inhibit IL-2 and interferon-gamma expression [48]. As an anti-inflammatory cytokine, IL-10 participates in the development of various diseases, such as chronic infection, kidney disease, cancer, and cardiovascular diseases [49]. Didion et al. have described a critical role of IL-10 in modulating endothelial function in hypertension [50]. It inhibits the production of pro-inflammatory cytokines via inhibiting T-helper1 (Th1) lymphocytes and stimulating B lymphocytes and Th2 lymphocytes and thus down-regulating the inflammatory response [51]. Moreover, IL-10 plays a role in the inhibition of cell adhesion molecules, tissue factor, fibrinogen, monocyte chemotactic protein-1, matrix metalloproteinase-9, inducible nitric oxide synthase, T-lymphocyte granulocyte-macrophage colony-stimulation factor, and smooth muscle cell proliferation [52]. Furthermore, a potent ability of IL-10 to suppress TNF-α and IL-1β production ensures its role as one of the most important immunoregulators as well as a mediator of inflammatory process [53]. In line with these previous reports, our present study confirmed that the model group IL-10 was significantly reduced, and after four weeks of XJEK and fosinopril treatment the reduction of IL-10 levels could be inhibited markedly.

## Conclusions

The present findings verify that XJEK ameliorates the post MI pathological alterations. Its action may be due to its role in enhancing ED function, oxidative stress and maintaining the immune balance. Therefore, XJEK may be used hopefully in clinical practice in the treatment of MI and other cardiovascular diseases.

## Additional files

Additional file 1: Figure S1. Representative figures of myocardial infarct area with TTC staining in MI mice (JPEG 4 kb)

Additional file 2: Figure S2. Changes of cardiac function in the four time point during an 8-week period (JPEG 36 kb)

## Abbreviations

±dp/dt_max_: Maximal rate of left ventricular systolic and diastolic pressure
Ang II: Angiotensin II
APD: Action potential duration
AR: Aorta radius
ASBP: Artery systolic blood pressure
AT_1_R: Ang II type 1 receptor
BZ: Border zone
CSA: Cross-sectional area
CVF: Collagen volume fraction
ECG: electrocardiography
ED: Endothelial dysfunction
ELISA: Enzyme linked immunosorbent assay
eNOs: Endothelial NO synthase
HE: Hematoxylin and eosin
HR: Heart rate
HW/BW: Heart weight/body weight
IFN-α: Interferon-gamma
IL-10: Interleukin-10
IL-1β: Interleukin-1β
LDA: Left anterior descending
LV: Left ventricle
LVEDP: Left ventricular end-diastolic pressures
LVSP: Left ventricular systolic pressures
MDA: Malondialdehyde
MI: Myocardial infarction
MMPs: Matrix metalloproteinases
MT: Media thickness
NADPH: Nicotinamide adenine dinucleotide phosphate
NO: Nitric oxide
OS: Oxidative stress
PBS: Phosphate-buffered saline
PVCA: Perivascular circumferential collagen area
ROS: Reactive oxygen species
SOD: Superoxide dismutase
TAA: Total aorta area
TBARS: the Thiobarbituric acid reactive substances
TNF-α: Tumor necrosis factor-α
TTC: Triphenyltetrazolium chloride
VG: Van Gieson
XJEK: Xinji’erkang
