# Correction: Protective effects of Xinji′erkang on myocardial infarction induced cardiac injury in mice

**DOI:** 10.1186/s12906-023-03865-5

**Published:** 2023-03-04

**Authors:** Juan Hu, Yong-xue Zhang, Li Wang, Ling Ding, Guang-yao Huang, Guo-wei Cai, Shan Gao

**Affiliations:** Department of Pharmacology, Basic Medical College, Anhui Medical University, Hefei, 230032 China

**Correction: BMC Complement Med Ther 17, 338 (2017)**

**https://doi.org/10.1186/s12906-017-1846-5**

Following publication of the original article [1], the authors identified an error in Fig. 3. The b4 of Fig. 3 in this published paper was wrongly used. The correct figure is given below.

**Fig. 3:**
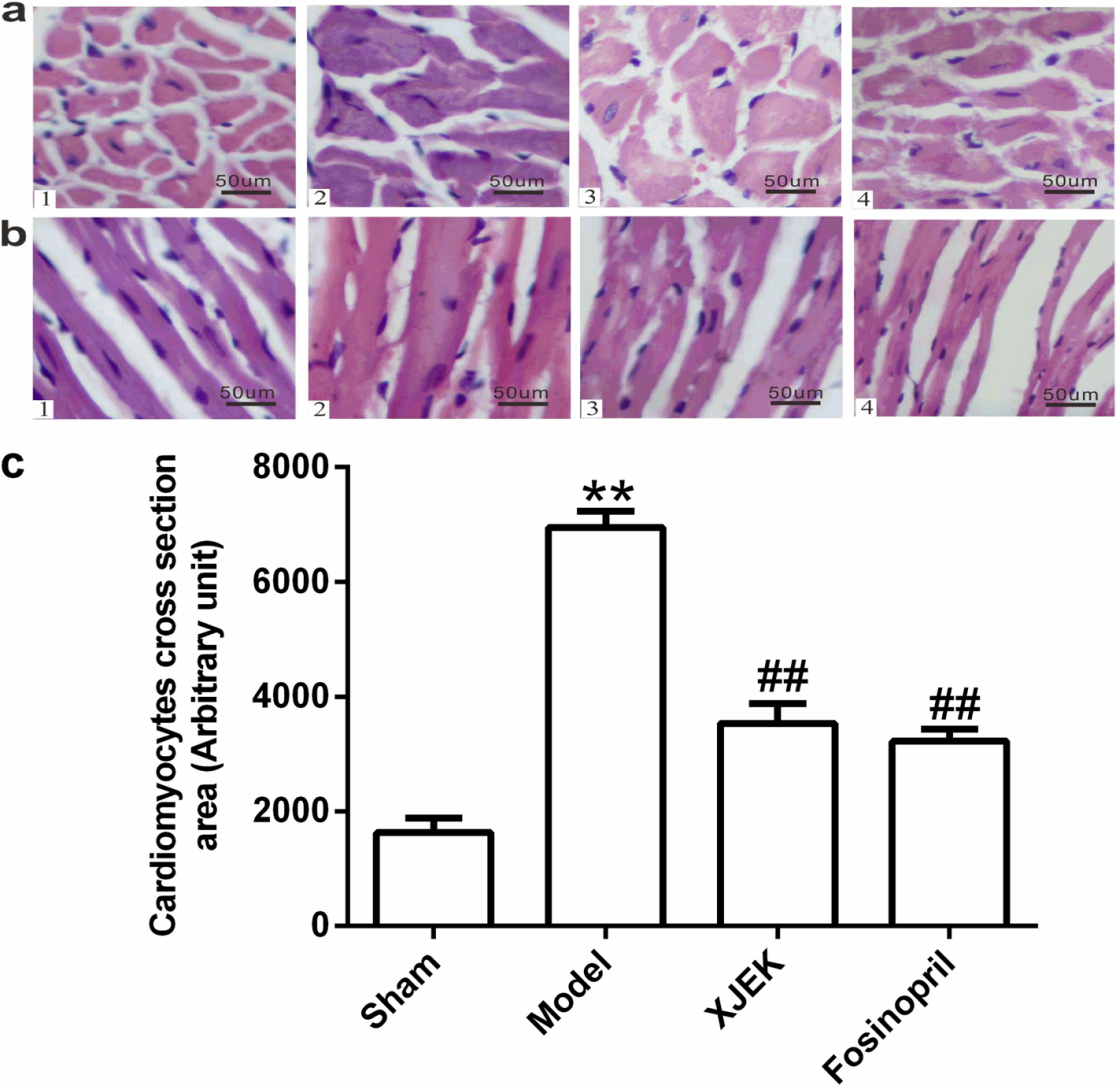
Effect of XJEK on cardiomyocyte CSA and cardiomyocyte long axis of MI mice (HE stain, magnification×400). **a** Representative images of histological section of cardiomyocyte cross-section (HE staining, magnification×400); **b** Representative images of histological section of cardiomyocyte long axis (HE staining, magnification×400); **c** Quantitative analyses results (mean ± SEM, *n*=6–9). (1) Sham group; (2) Model group; (3) XJEK group; (4) Fosinopril group. ^*^ *P*<0.05, ^**^ *P*<0.01 *vs*. Sham group; ^*#*^*P*<0.05, ^*##*^*P*<0.01 *vs.* Model group

The original article [1] has been corrected.
