# Supplementary figures and images for: Protective effects of Xinji′erkang on myocardial infarction induced cardiac injury in mice
Source: BMC Complement Altern Med. 2017 Jun 26;17:338. doi: 10.1186/s12906-017-1846-5 (PMC5485507; doi:10.1186/s12906-017-1846-5)

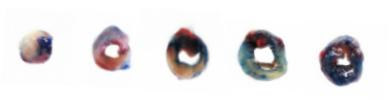

Supplement: Supplementary file 1 — Representative figures of myocardial infarct area with TTC staining in MI mice (JPEG 4 kb) [file 12906_2017_1846_MOESM1_ESM.jpg]

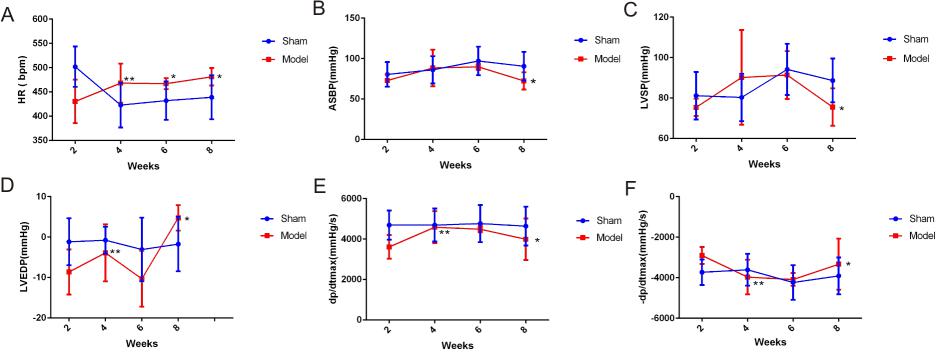

Supplement: Supplementary file 2 — Changes of cardiac function in the four time point during an 8-week period (JPEG 36 kb) [file 12906_2017_1846_MOESM2_ESM.jpg]
